# Is minimally invasive superior than open transforaminal lumbar interbody fusion for single-level degenerative lumbar diseases: a meta-analysis

**DOI:** 10.1186/s13018-018-0941-8

**Published:** 2018-09-20

**Authors:** Aimin Li, Xiang Li, Yang Zhong

**Affiliations:** Department of Orthopedics, The 5th Central Hospital of Tianjin, Tianjin, 30000 People’s Republic of China

**Keywords:** Single-level, Degenerative lumbar disease, Transforaminal lumbar interbody fusion, Minimally invasive

## Abstract

**Background:**

In recent years, the minimally invasive transforaminal lumbar interbody fusion (MI-TLIF) is increasingly used to manage the lumbar degenerative disease. However, whether MI-TLIF was superior than open transforaminal lumbar interbody fusion (O-TLIF) was controversial. The aim of this meta-analysis was to compare the clinical outcomes between the MI-TLIF and O-TLIF in single-level degenerative lumbar diseases.

**Methods:**

Two reviewers independently searched EMBASE, PubMed, Web of Science, and Google database from inception to February 2018 for studies comparing the MI-TLIF and O-TLIF approach for single-level lumbar degenerative disease. The data were extracted and analyzed for primary outcomes such as total blood loss, visual analog score (VAS), and other secondary outcomes (length of hospital stay, operation time, fluroscopic time, and Oswestry Disability Index (ODI)). Meta-analysis was performed by Stata 12.0.

**Results:**

Seven RCTs were finally included in this meta-analysis. Compared with O-TLIF, MI-TLIF was associated with significantly less blood loss (weighted mean difference (WMD) = − 291.46; 95% confidence interval (CI) − 366.66 to − 216.47; *P* = 0.000,). There was no significant difference between the length of hospital stay, postoperative VAS, and ODI. Compared with O-TLIF, MI-TLIF was associated with an increase of the fluroscopic time (*P* < 0.05).

**Conclusion:**

The MI-TLIF showed significantly less blood loss compared with O-TLIF and more fluroscopic time. There was no significant difference between the length of hospital stay, postoperative VAS, and ODI. More high-quality studies and subsequent meta-analyses are needed in the future.

## Background

Minimally invasive transforaminal lumbar interbody fusion (MI-TLIF) was first introduced by Harms and Rolinger in the year of 1982 [1]. MI-TLIF has been used increasingly in lumbar degenerative diseases for decades based on the shorter length of hospital stay and blood loss than open TLIF (O-TLIF) [2–4]. O-TLIF requires wide dissection of the sacral spinalis, which causes severe soft tissue injuries and more perioperative blood loss [5].

O-TLIF was also associated with residual back pain for some patients after surgery [6, 7]. As a new technique, MI-TLIF exposes spinal joints and transverse processes directly through the space between sacrospinous muscles. Several studies show that MI-TLIF could reduce the injury of the iatrogenic soft tissue caused by muscle stripping and traction in surgery [8]. However, the MI-TLIF has some drawbacks. During the minimally invasive procedure, only one side of the articular process was removed. For patients with severe spinal stenosis, the central tube decompression could not be performed.

All of what mentioned above limited the application of this technique in such patients. The potential benefits of MI-TLIF versus O-TLIF for single-level lumbar degenerative diseases remain controversial.

The aim of the current meta-analysis was to assess the efficacy and safety of O-TLIF and MI-TLIF for single-level degenerative lumbar diseases in terms of total blood loss, the length of hospital stay, operation time, postoperative visual analog scale (VAS), and Oswestry Disability Index (ODI).

## Methods

This study was undertaken in accordance with the Preferred Reporting Items for Systematic Reviews and Meta-Analyses (PRISMA) statement [9]. Ethical approval and written informed consent from patients were not necessary because our study was based on summaries and analyses of results of existing studies.

### Search strategy

Two reviewers (Aimin Li and Yang Zhong) independently searched the EMBASE, PubMed, Web of Science, and Google database from inception to February 2018. Search core terms included “transforaminal lumbar interbody fusion,” “TLIF,” “minimally invasive spine surgery,” “MIS,” “minimally invasive,” and “clinical outcome.” We searched with the restricted language of English and only included articles published in the English language. Two reviewers (Aimin Li and Xiang Li) also manually searched the references of each retrieved papers for additional studies.

### Study selection

A study was considered qualified for inclusion if studies were randomized controlled trials (RCTs) or no-RCT study, comparing MI-TLIF with O-TLIF for single-level degenerative lumbar diseases. And all studies reported data for at least one of the following index: total blood loss, length of hospital stay, operation time, fluroscopic time, postoperative VAS, and ODI.

Following studies were excluded from the meta-analysis: conference abstracts; unpublished studies; case report; studies not suitable with the inclusive criteria; duplicated reports; multi-level lumbar degenerative disease samples; samples of fracture, infection, tumor, or postoperative recurrence; and samples of severe osteoporosis and other metabolic diseases.

### Study selection

All potentially relevant studies were imported into Endnote Software (Version X7, Thompson Reuters, CA, USA) and duplicated studies were excluded. Then, the two researchers who had searched the databases excluded studies based on titles and abstracts. The two researchers read the full text of the remaining studies and excluded those that were not suitable with the inclusive criteria. Disagreements were solved by checking the articles and contacting related authors if required.

### Data extraction

The following data were extracted by two researchers independently from the final eligible studies. Extracted information includes first author, publication year, country, study design, and numbers of cases and controls. Data were collected on the following primary outcomes: estimated blood loss and visual analog score. Data were also collected on the following secondary outcomes: operative time, length of hospital stay, or ODI.

### Quality assessment

The quality of each included study was assessed on the basis of the Cochrane Handbook for Systematic Reviews of Interventions, version 5.1.0 (http://handbook.cochrane.org/) [10]. It included the following sections: random sequence generation, allocation concealment, blinding of participant and personnel, blinding of outcome assessment, incomplete outcome data, selective reporting, and other bias. Each domain was measured as low bias, unclear bias, or high bias. Diversities between assessments by the two reviewers were resolved by discussion.

### Statistical analysis

The extracted outcomes from data could not be compared directly because the included studies were comprehensive. Outcomes for which sufficient, equivalent data were meta-analyzed using Forest plots generated with Stata 12.0. We analyzed continuous data using weighted mean differences (WMD) and their 95% confidence interval (CI), such as estimated blood loss, operative time, length of hospital stay, VAS, and ODI. Analyses were undertaken if at least three studies comparing the same outcome for MI-TLIF and O-TLIF could be combined. Each meta-analysis was done using all available searches. Pooled results were assessed for heterogeneity using the *I*^2^ tests. Heterogeneity was defined as good when *I*^2^ was between 0 and 25%; low, between 25.1 and 50%; moderate, between 50.1 and 75%; or high, between 75.1 and 100%. Fixed-effects meta-analysis was performed when *P* ≥ 0.1 and *I*^2^ ≤ 50%; otherwise, random-effects meta-analysis was performed. Sensitivity analysis was done for all outcomes to analyze the reason for the formation of heterogeneity. All statistical analyses were undertaken using Stata 12.0. Additional “leave-one-out” sensitivity analyses will be performed to explore whether the results were dominated by a single study, which was investigated by omitting one study at each turn and examining the influence of each individual study on the final outcomes. Publication bias was performed by funnel plot and Begg’s test. If the effect size was symmetrical and *P* value drawn from Begg’s test was more than 0.05 indicated that there was no publication bias.

## Results

### Search results

**Fig. 1:**
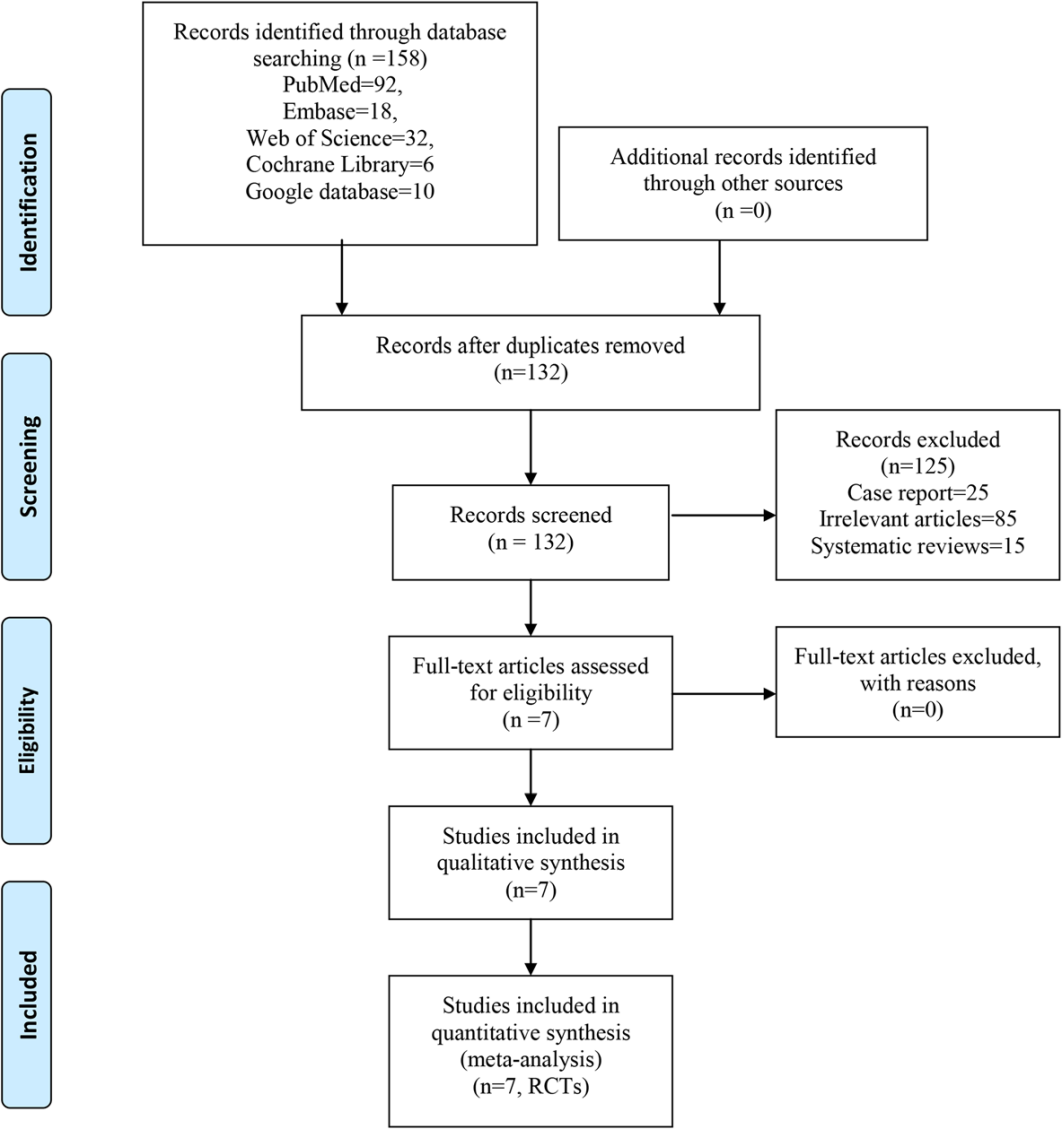
Flow of trials through the meta-analysis

After systematic search of the electronic databases, a total of 363 studies were identified, and no additional records were found during manual searches of review articles and relevant publications. After removing 244 duplicate studies using Endnote X7 (Version X7, Thompson Reuters, CA, USA), another 589 studies were excluded based on their titles and abstracts. The remaining 30 studies were read in full, and 20 were excluded because they failed to satisfy the selection criteria. In the end, 7 studies were included in this meta-analysis [11–17]. Flow of trials through the meta-analysis can be seen in Fig. 1.

### General characteristics of the included studies

The general characteristics of the included studies are listed in Table 1. The publication year of the included studies ranged from 2010 to 2017. The sample size ranged from 23 to 72. Follow-up duration ranged from 6 to 60 months.

**Table 1:** The general characteristic of the included studies

Reference	Country	Mean age (years)	Sample size	Study type	Follow-up	O-TLIF	MI-TLIF
Singh et al. [11]	USA	51.67/49.85	33/33	RCTs	6 months	Midline incision	Unilateral approach
Kulkarni et al. [12]	India	51.55/50.40	36/25	RCTs	36.5 months	Posterior midline incision	Unilateral approach
Lee et al. [13]	Singapore	56.6/ 56.8	40/40	RCTs	24 months	NS	NS
Seng et al. [14]	Singapore	52.2/56.6	72/72	RCTs	60 months	Midline incision	Parasagittal incision
Serban et al. [15]	USA	51.3/ 50.1	24/23	RCTs	6 months	Midline skin	Parasagittal incision
Wang et al. [16]	China	56.4/ 54.2	42/39	RCTs	36.1 months	Midline open approach	NS
Wang et al. [17]	China	47.9/53.2	42/43	RCTs	26.3 months	Midline open approach	Unilateral approach

*RCTs*, randomized controlled trials, *MI-TLIF*, minimally invasive transforaminal lumbar interbody fusion, *O-TLIF*, open transforaminal lumbar interbody fusion, *NS* not stated

### Risk of bias of the included studies

**Fig. 2:**
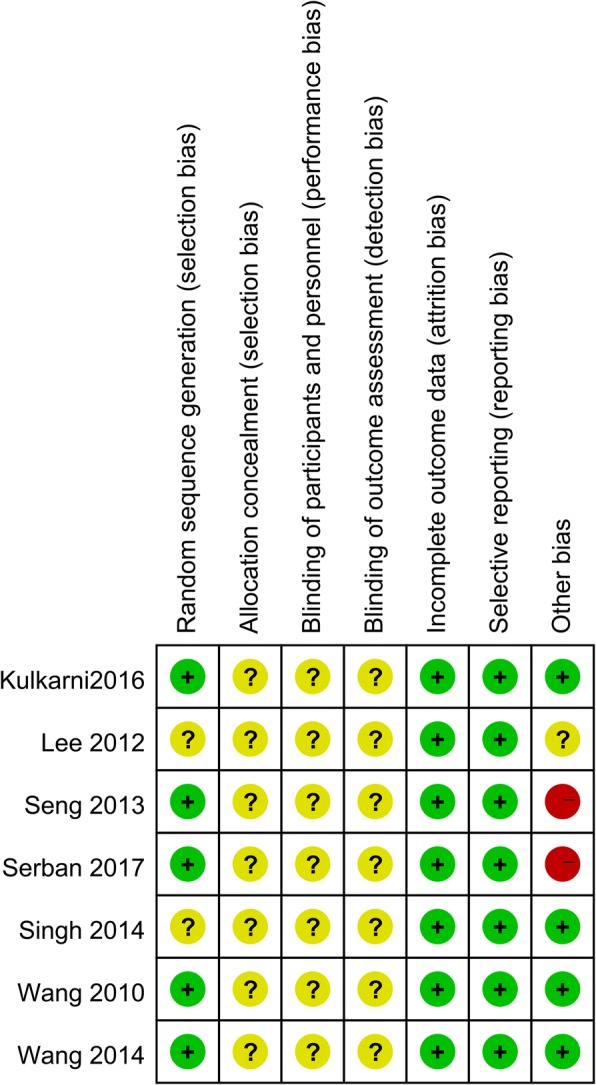
The risk of bias summary, +, no bias; −, bias; ?, bias unknown

**Fig. 3:**
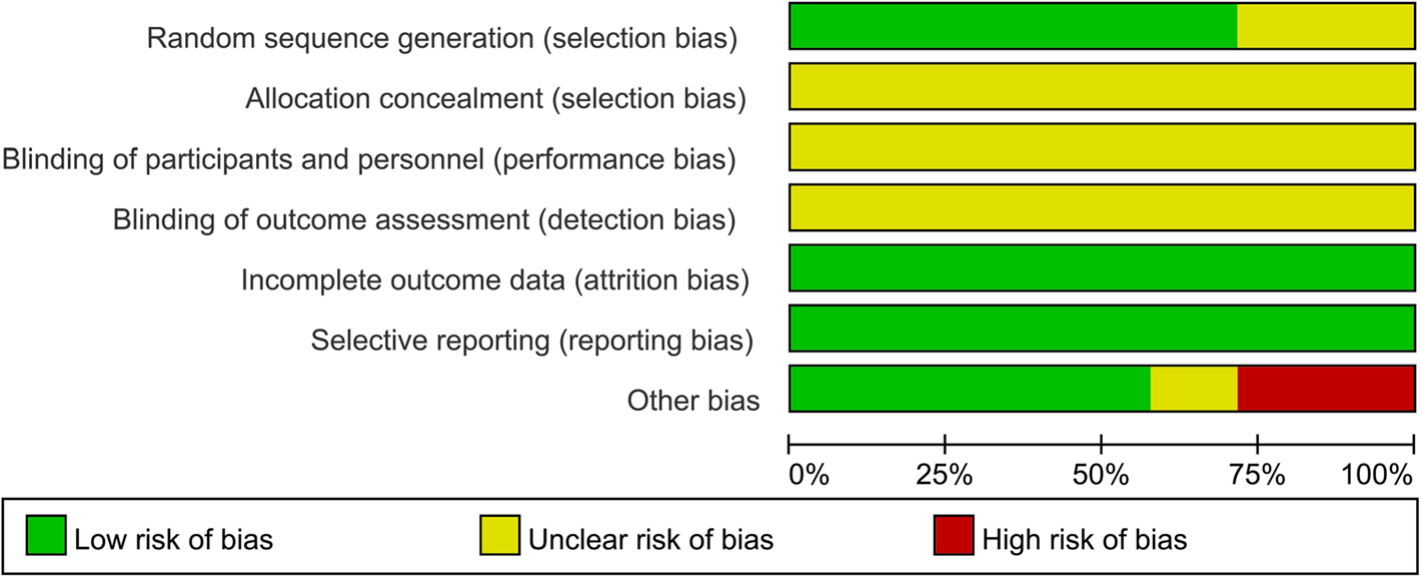
Risk of bias of summary of the included randomized controlled trials

Risk of bias summary and risk of bias graph can be seen in Figs. 2 and 3 respectively. Five studies performed proper random sequence generation, and the rest two studies did not perform proper random sequence generation and were listed as unclear risk of bias. Since these two operations could not be performed blinded, thus the blinding to the participants and personnel was all with unclear risk of bias.

### Results of meta-analysis

#### Total blood loss

**Fig. 4:**
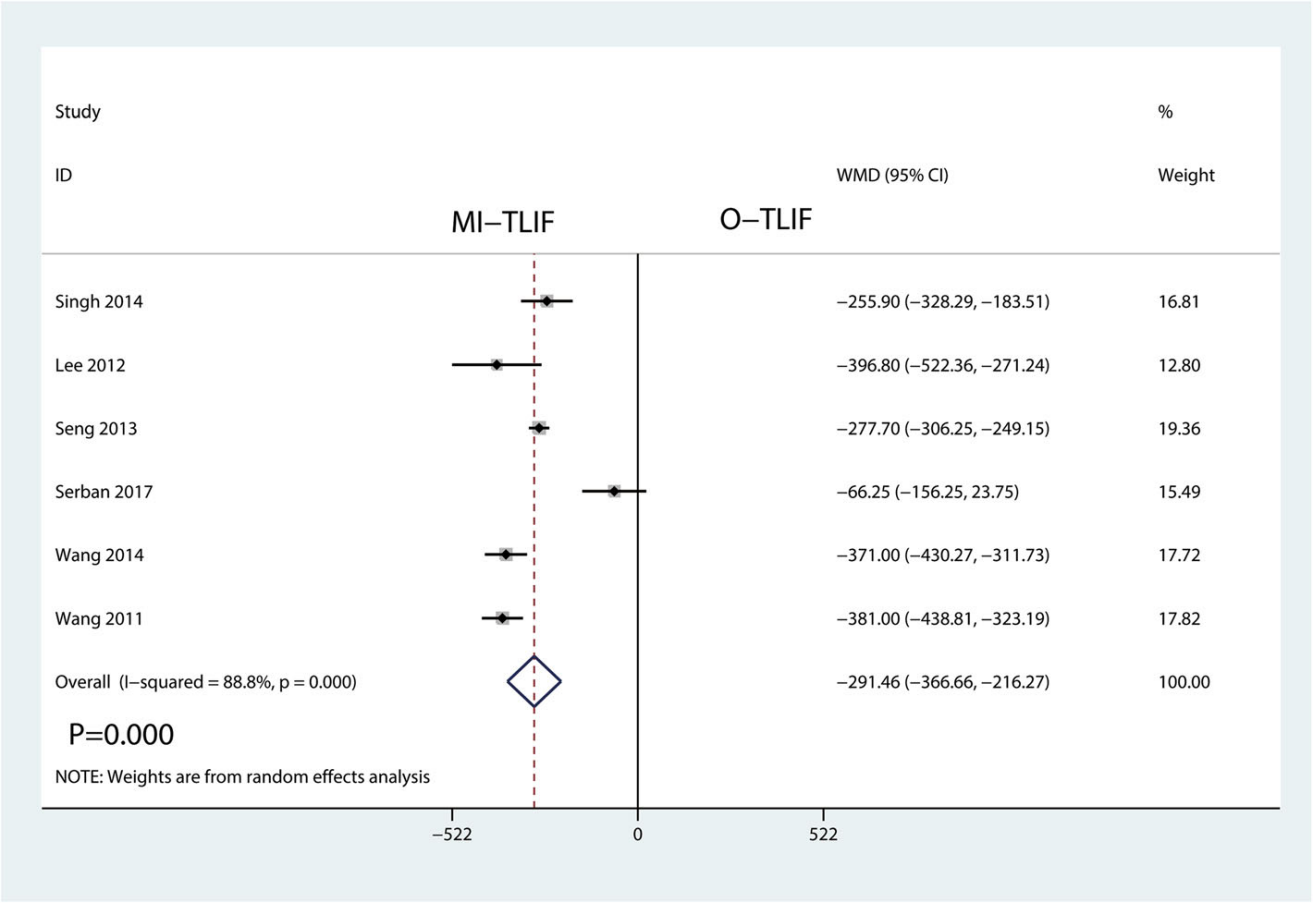
Forest plots of the included studies comparing the total blood loss

Seven studies including 532 patients investigated the total blood loss of two approaches. Meta-analysis showed that MI-TLIF was associated with a reduction of the total blood loss than O-TLIF (WMD = − 291.46; 95% CI − 366.66 to − 216.47; *P* = 0.000; Fig. 4**)**. This meta-analysis involved a random-effects model because of high heterogeneity among the studies (*I*^2^ = 88.8%).

#### Length of hospital stay

**Fig. 5:**
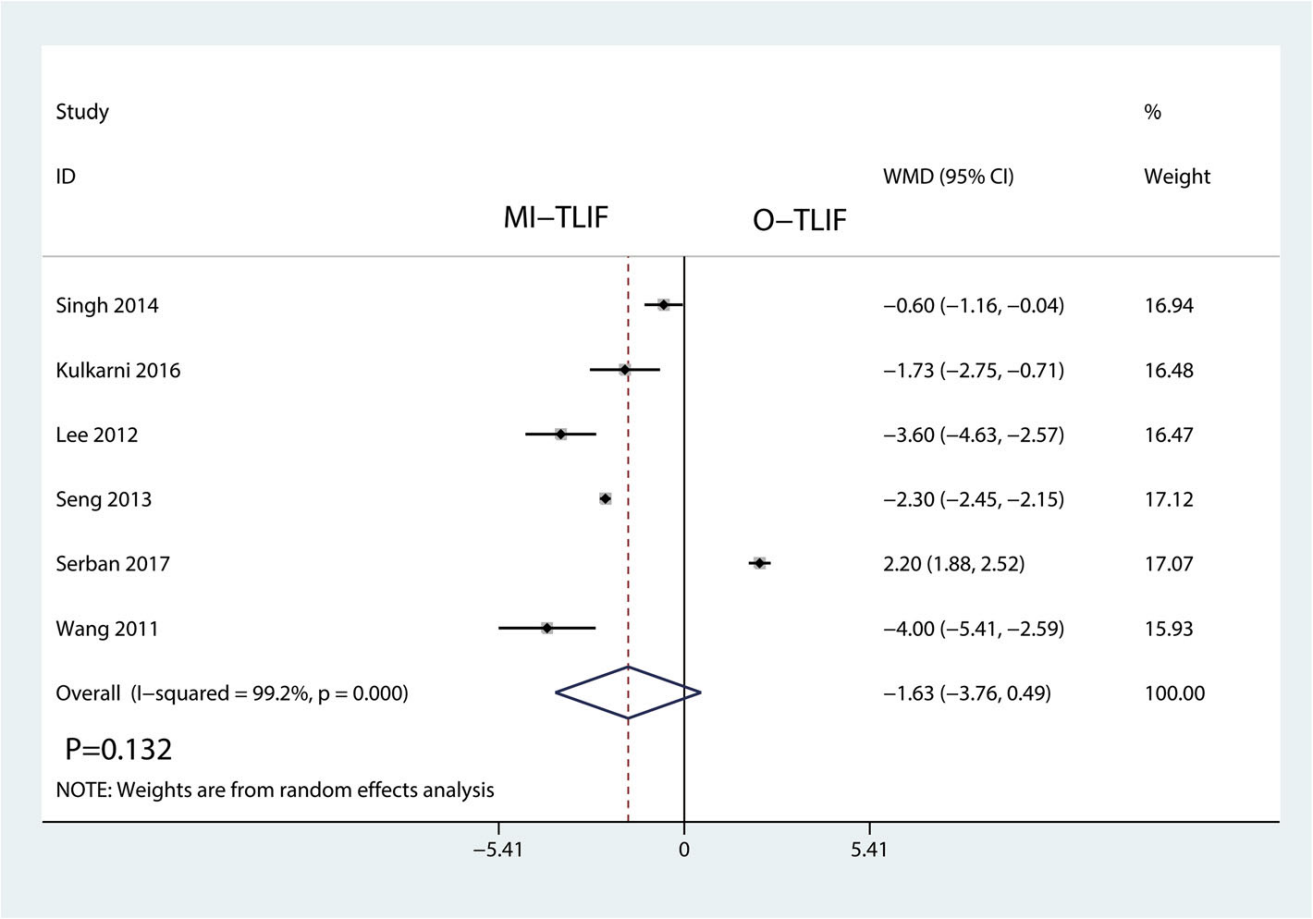
Forest plots of the included studies comparing the length of hospital stay

Six studies including 522 patients reported data on the length of hospital stay. This meta-analysis showed that there was no significant difference between the MI-TLIF and O-TLIF in terms of the length of hospital stay (WMD = − 1.63, 95% CI − 3.76 to 0.49; *P* = 0.132; Fig. 5). Meta-analysis involved a random-effects model because of high heterogeneity among the studies (*I*^2^ = 99.2%).

#### Operating time

**Fig. 6:**
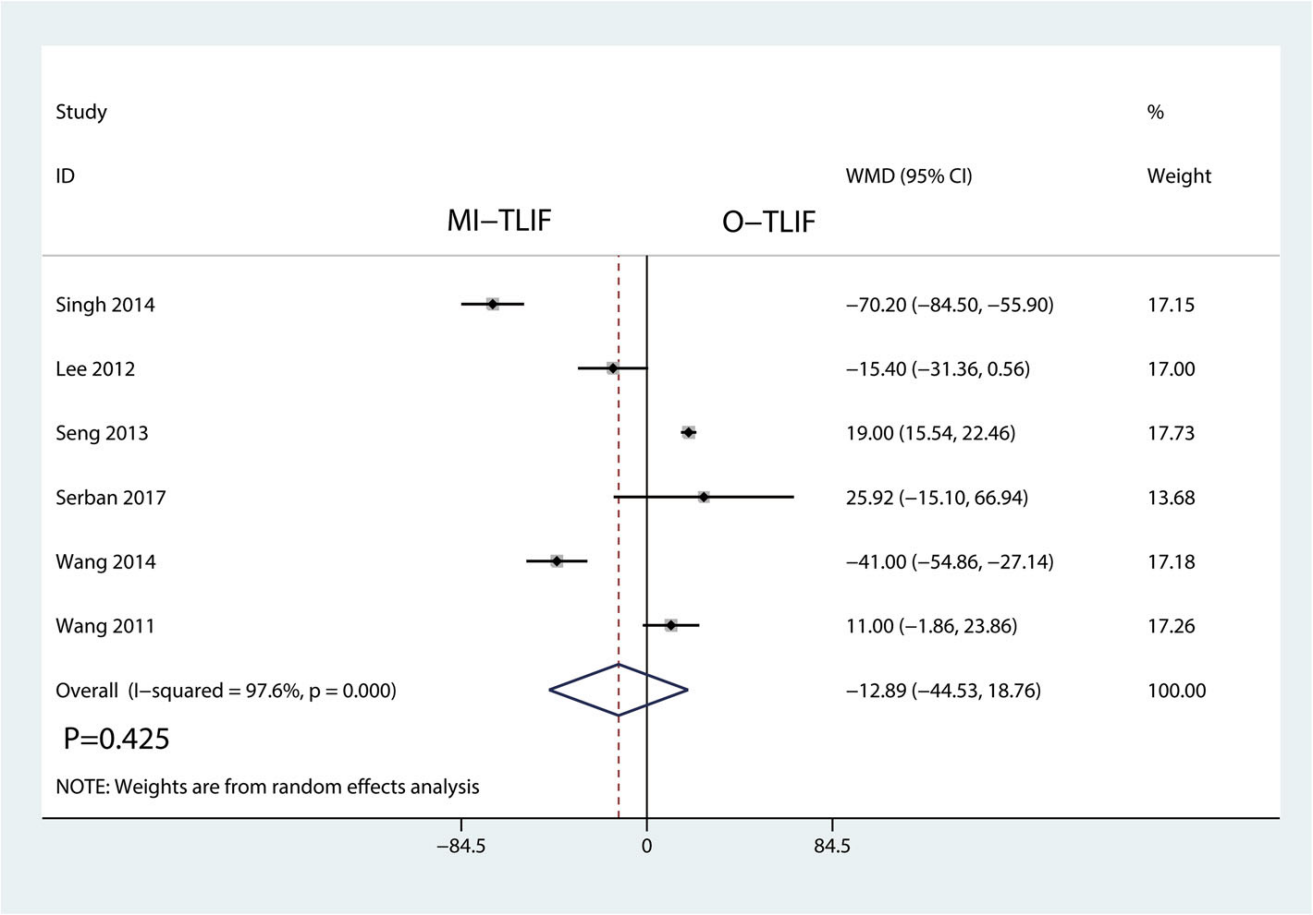
Forest plots of the included studies comparing the operating time

Six studies including 532 reported data on operating time. Meta-analysis result showed that there was no significant differences for operating time in MI-TLIF group and O-TLIF group (WMD = − 12.89, 95% CI − 44.53 to 18.76; *P* = 0.425; Fig. 6). This meta-analysis involved a random-effects model because of high heterogeneity among the studies (*I*^2^ = 97.6%).

#### Postoperative VAS score

**Fig. 7:**
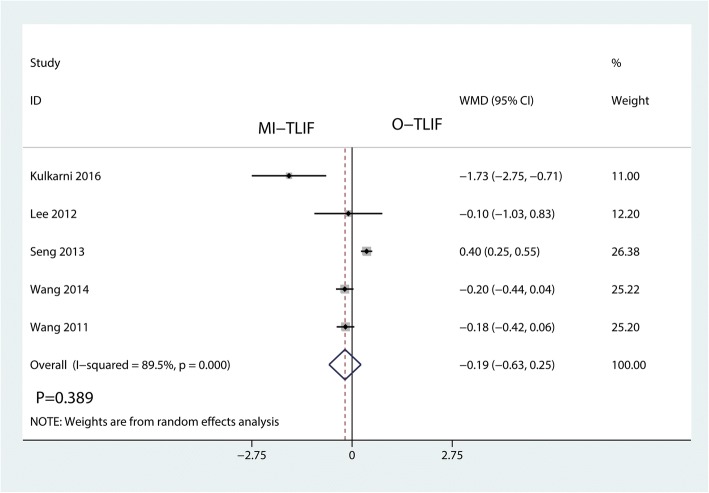
Forest plots of the included studies comparing the postoperative VAS score

Five studies involving 457 patients investigated the outcome of postoperative VAS. This meta-analysis showed that there was no significant difference between the postoperative VAS after MI-TLIF and O-TLIF (WMD = − 0.19, 95% CI − 0.63 to 0.25; *P* = 0.389; Fig. 7). This meta-analysis involved a random-effects model because of high heterogeneity among the studies (*I*^2^ = 88.8%).

#### Postoperative ODI

**Fig. 8:**
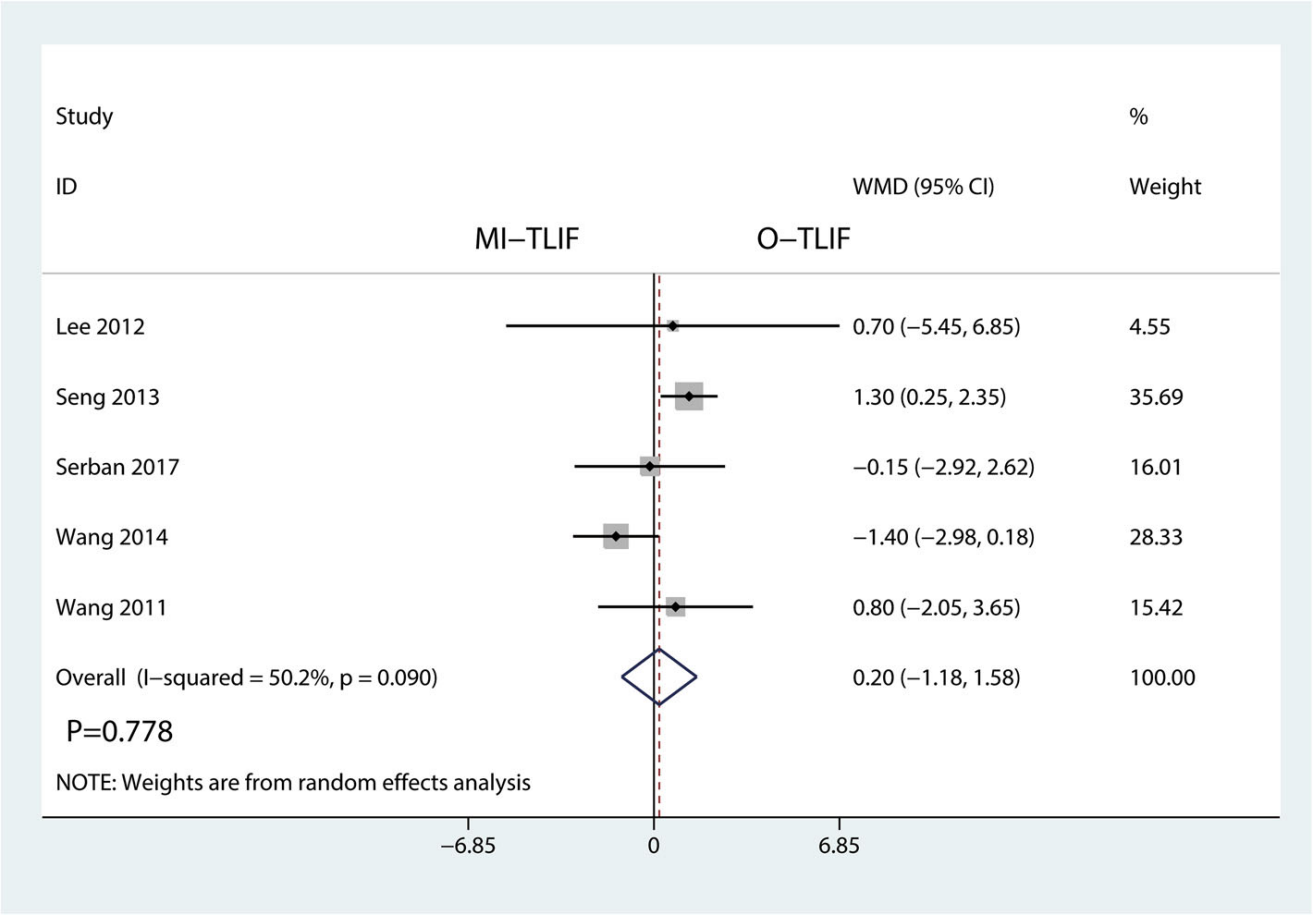
Forest plots of the included studies comparing the postoperative ODI

Five studies investigated the outcome of postoperative ODI. This meta-analysis showed that there was no significant difference between the ODI between MI-TLIF and O-TLIF groups (WMD = 0.20, 95% CI − 1.18 to 1.58; *P* = 0.778; Fig. 8). This meta-analysis involved a random-effects model because of no significant heterogeneity among the studies (*I*^2^ = 50.2%).

#### Fluroscopic time

**Fig. 9:**
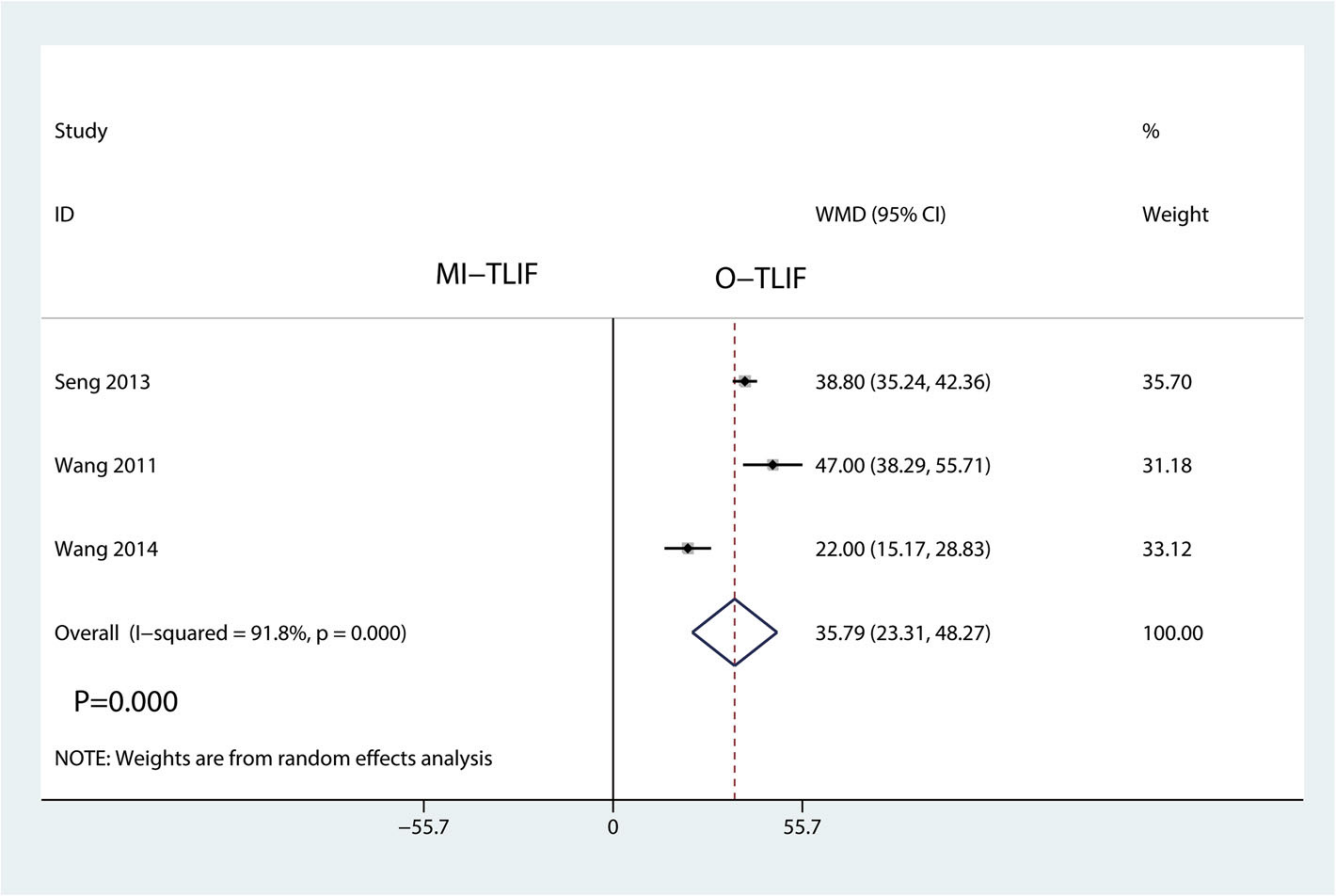
Forest plots of the included studies comparing the fluroscopic time

Three studies investigated the outcome of fluroscopic time. This meta-analysis showed that MI-TLIF was associated with an increase of the fluroscopic time than O-TLIF (WMD = 35.79, 95% CI 23.31 to 48.27; *P* = 0.000; Fig. 9). This meta-analysis involved a random-effects model because of no significant heterogeneity among the studies (*I*^2^ = 91.8%).

#### Sensitivity analysis and publication bias

**Fig. 10:**
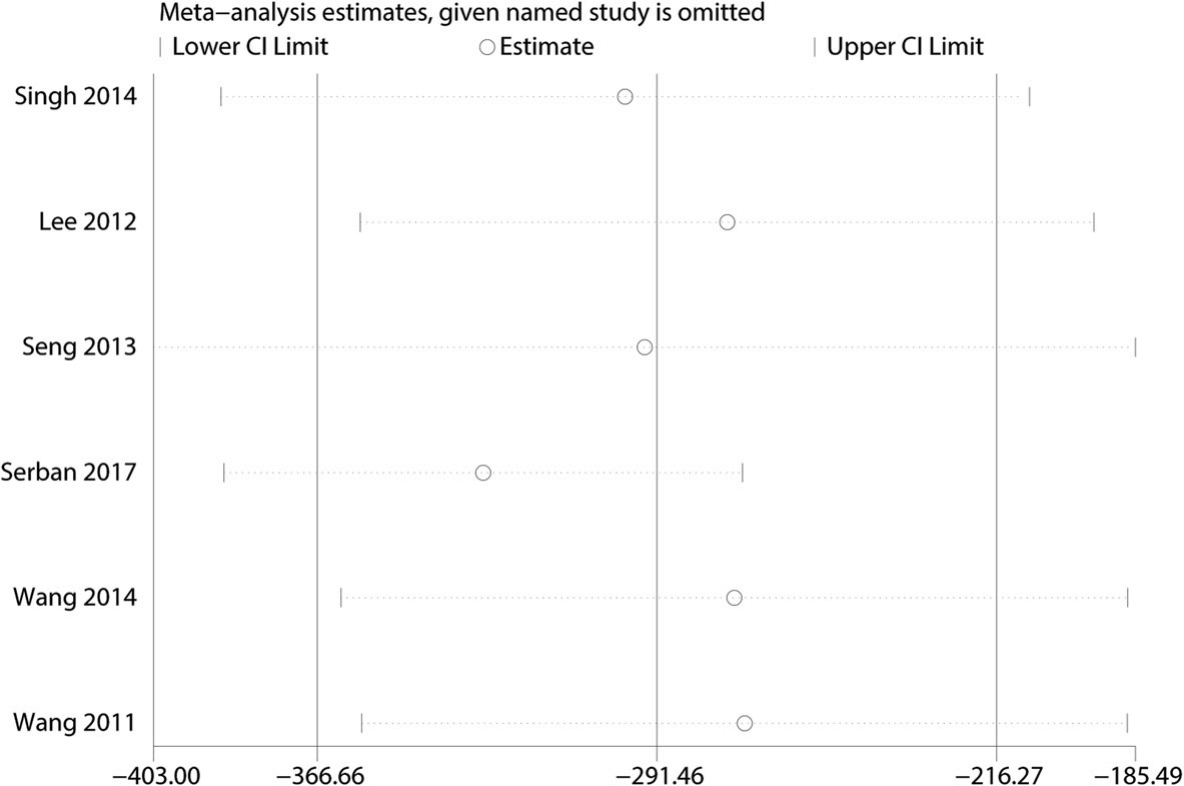
Sensitivity analysis of the total blood loss

**Fig. 11:**
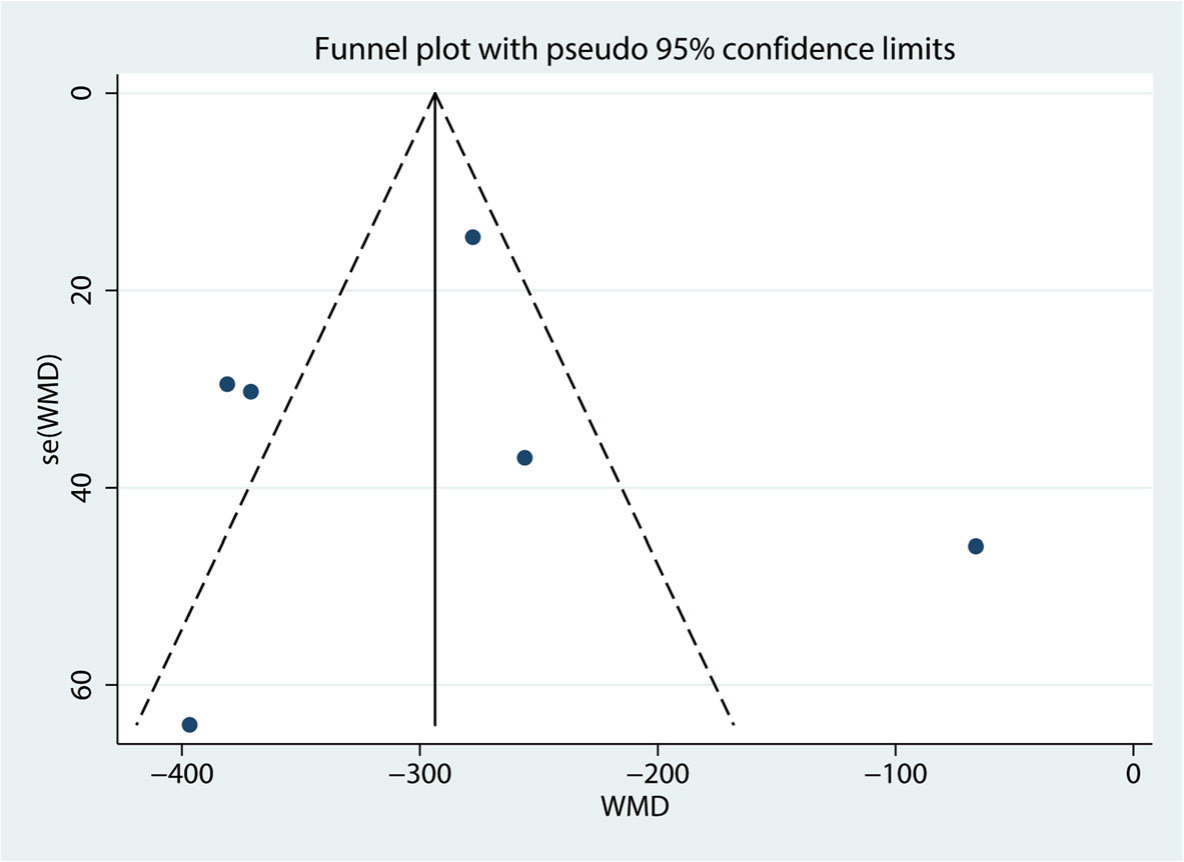
Funnel plot of the total blood loss

**Fig. 12:**
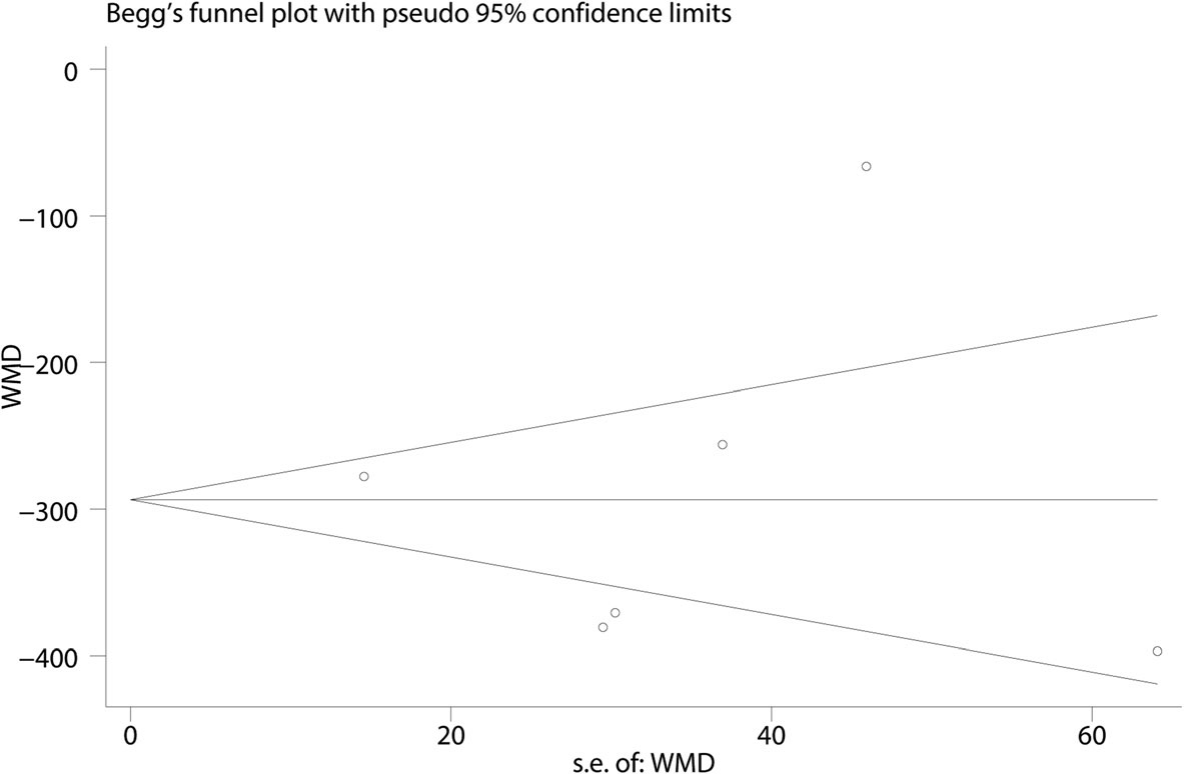
Begg’s test for total blood loss

Additional leave-one-out sensitivity analyses show that the overall effects were not altered and thus the results were relatively robust (Fig. 10). Publication bias was measured by funnel plot and the effects size was symmetrical; thus, there was no publication bias for total blood loss (Fig. 11). Then, Begg’s test was performed and the *P* value was 0.985 indicating that there was no publication bias (Fig. 12).

## Discussion

### Main finding

Our meta-analysis comprehensively and systematically reviewed the current available literature and found that (1) MI-TLIF have experienced less intraoperative blood loss than those in O-TLIF patients; (2) there was no significant difference between the length of hospital stay, operating time, postoperative ODI, and VAS; (3) and MI-TLIF was associated with an increase of the fluroscopic time than O-TLIF.

### Comparison with other meta-analyses

Several meta-analyses on the topic have been published [18–20]. Although the main finding of our meta-analysis was consistent with previous meta-analyses, differences between ours and the previous ones should be noted. First, these previous meta-analyses included retrospective comparison studies and thus may cause large selection bias. In comparison, our current meta-analysis only included prospective observe studies and thus could avoid this bias. Second, we also evaluated the effect of fluroscopic time between the two methods and provided a new insight into the advantage of these two methods.

This meta-analysis is the latest one to evaluate the efficacy and safety of MI-TLIF and O-TLIF for single-level degenerative lumbar diseases and to determine which surgical technique is superior. A few studies [12, 13] have described a superior or comparative outcome for patients undergoing MI-TLIF approach for single-level degenerative lumbar diseases. Singh et al. [11] showed MI-TLIF demonstrated reductions of operative time, length of stay, anesthesia time, VAS scores, and estimated blood loss compared with the open method. Lee et al. [5] verified the safety of MI-TLIF approach and similar operative duration, good clinical outcomes. Wang et al. [16] demonstrated that minimally invasive TLIF has similar surgical efficacy with the traditional open TLIF in treating one-level degenerative lumbar diseases. Serban et al. [15] justified the two techniques provided similar clinical and radiological outcomes. Seng et al. [14] showed MI-TLIF is comparable with open TLIF in terms of midterm clinical outcomes and fusion rates.

Furthermore, a meta-analysis carried out in 2015 concluded that MI-TLIF is associated with decreased complication rates, and increased radiation exposure [21]. Current meta-analysis indicated that MI-TLIF was associated with an increase of the radiation exposure than O-TLIF. And the conclusion was consistent with previous meta-analysis. The two approaches for single-level degenerative lumbar diseases appear to be associated with similar results on operative time and postoperative ODI. Due to the limited number of studies available, we did not investigate other clinical outcomes such as risk of complications and fusion rates.

Theoretically, many doctors prefer performing MI-TLIF because it may be associated with small incisions and more rapid recovery. However, the learning curve for MI-TLIF was always longer than O-TLIF. Park et al. [22] revealed that perioperative complications occurred more often in the early period of a surgeon’s experience with MI-TLIF. Thus, MI-TLIF need for more learning time than O-TLIF. Nandyala et al. [23] identified that MI-TLIF is a technically difficult procedure to the practicing spine surgeon and further studies are warranted to delineate the methods to minimize the complications associated with the learning curve.

There were several limitation of current meta-analysis: (1) there was a large heterogeneity between the included outcomes, though we performed a sensitivity analysis; however, we could not found the heterogeneity. (2) There were some missed data and we could not obtained the original data and thus may cause the bias. (3) The number of enrolled patients was relatively small, which may limit the statistical effects of the data. (4) Follow-up in some studies were relatively short and long-term effects of O-TLIF versus MI-TLIF need further study.

## Conclusion

In conclusion, this meta-analysis suggests that MI-TLIF appeared to achieve less blood loss than O-TLIF. However, the two procedures, MI-TLIF and O-TLIF, were comparable in clinical efficacy and fusion rates. Due to the limited number of the included RCTs, more well-designed multicenter RCTs with larger sample sizes and long-term follow-up are still needed to compare the clinical efficacy and safety of MI-TLIF and O-TLIF.

## Abbreviations

df: Degrees of freedom
fixed: Fixed-effects modeling
IV: Inverse variance
MI-TLIF: Minimally invasive transforaminal lumbar interbody fusion
ODI: Oswestry Disability Index
O-TLIF: Open transforaminal lumbar interbody fusion
random: Random-effects modeling
RCTs: Randomized controlled trials
SD: Standard deviation
TLIF: Transforaminal lumbar interbody fusion
VAS: Visual analog score
